# The complete chloroplast genome sequence of *Liparis japonica* (Orchidaceae)

**DOI:** 10.1080/23802359.2019.1636728

**Published:** 2019-07-12

**Authors:** Jianfang Li, Qian Yang, Zhan-Lin Liu

**Affiliations:** Key Laboratory of Resource Biology and Biotechnology in Western China (Ministry of Education), College of Life Sciences, Northwest University, Xi'an, China

**Keywords:** *Liparis japonica*, Orchidaceae, cpDNA, phylogeny

## Abstract

The Orchidaceae contains numerous species with great ecological and economic values. In this study, the complete chloroplast genome of *Liparis japonica* was presented by next-generation sequencing technologies. The cpDNA is 152,084 bp in length with a large single copy region (LSC) of 85,398 bp, a small single copy region (SSC) of 14,774 bp, and a pair of inverted repeat regions (IRs) of 25,956 bp. It contains 132 genes, including 79 protein-coding genes, 38 tRNA genes, 8 rRNA genes, and 7 pseudogenes. The overall GC content is 37.0%, while the corresponding values in the LSC, SSC, and IR region are 34.3, 30.0, 43.5%, respectively. The phylogenetic analysis shows that *Liparis japonica* is sister to *Liparis loeselii* and the genus *Liparis* is closely related to *Dendrobium*.

The orchidaceae is a diverse and widespread family with about 25,000 species (Givnish et al. 2015). It is of great ecological and economic value for its diversified habits and morphological traits. All orchids are classified as Endangered plants and listed in the Convention on International Trade in Endangered Species of Wild Fauna and Flora (CITES). The phylogenetic relationships among Orchidaceae taxa are far from resolved due to the large scale of species number. *Liparis japonica*, widely distributed in East Asia, is a traditional Chinese medicine used to treat metrorrhagia, amygdalitis, and traumatic injury (Song et al. 2018). For the important horticultural value, its natural population sizes have dramatically declined for the over-exploitation and habitat destruction over the past decades. In this study, we determined the complete chloroplast (cp) genome of *Liparis japonica* by next-generation sequencing technology to provide new data for the phylogenetic analysis of Orchidaceae and conservation management of *Liparis japonica*.

Total genomic DNA was extracted from fresh leaves of *Liparis japonica* collected from Qinling Mountains, China (N33.54° E108.55°). The voucher (2016LIU128) is deposited at the Evolutionary Botany Laboratory (EBL), Northwest University. The genome DNA was sequenced by Biomarker Technologies Co, Ltd (Beijing, China) using the Illumina HiSeq 2500 platform. After trimming with NGSQC Toolkit_v.2.3.3 (Patel and Jain 2012), the clean reads were assembled by MIRA 4.0.2 (Chevreux et al. 2004) and MITOBIIM V1.8 (Hahn et al. 2013). The genome was annotated by Geneious R8.0.2 (Biomatters Ltd., Auckland, New Zealand) with *Dendrobium nobil* (KX377961) as reference.

The cpDNA of *Liparis japonica* (GenBank accession number MK886512) is 152,084 bp in length, with a large single copy region (LSC) of 85,398 bp and a small single copy region (SSC) of 14,774 bp separated by a pair of inverted repeat regions (IRs) of 25,956 bp. It contains 132 genes, including 79 protein-coding genes, 38 tRNA genes, 8 rRNA genes, of which 19 genes are duplicated in the IRs. Among the annotated genes, 12 (*rps16*, *atpF*, *rpoC1*, *petB*, *rpl2*, *ndhB*, *trnK-UUU*, *trnG-UCC*, *trnL-UAA*, *trnV-UAC*, *trnl-GAU*, *trnA-UGC*) harbor a single intron and three (*ycf3*, *rps12*, *clpP*) have two introns. The overall GC content is 37.0%, while the corresponding values in the LSC, SSC, and IR region are 34.3, 30.0, and 43.5%, respectively. Among the 11 *ndh* genes usually annotated in most of the angiosperms, only *ndhB* gene is found to encode functional *ndh* protein, while others are pseudogenes (*ndhA*, *ndhD*, *ndhE ndhF*, *ndhG*, *ndhH*, *ndhJ*) or physically lost.

Twenty publicly available cp genomes were used to construct the phylogenetic relationships among Orchidaceae taxa. The alignment was conducted by MAFFT 7.310 (Katoh and Standley 2016). The maximum likelihood (ML) tree was implemented by RAxML v 7.2.8 (Stamatakis 2006) with *Iris sanguinea* (NC_029227) and *Narcissus poeticus* (NC_039825) as outgroup. The phylogenetic tree indicates that *Liparis japonica* is sister to *Liparis loeselii* and the genus *Liparis* is closely related to *Dendrobium* as supported by the previous studies (Givnish et al. 2015, Krawczyk et al. 2018) (Figure 1).

**Figure 1.**
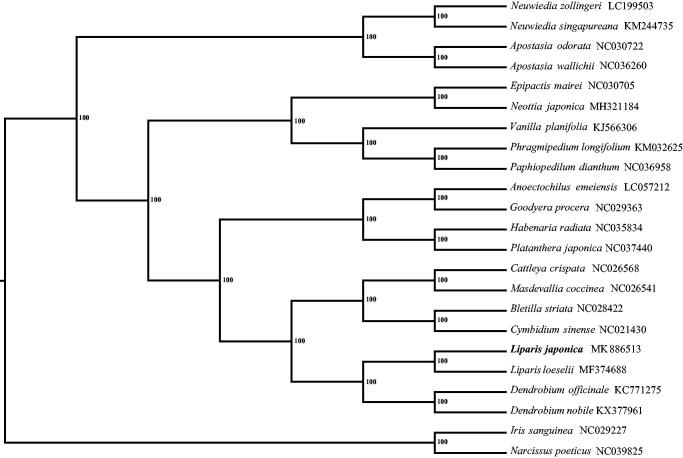
The phylogenetic tree constructed with 21 complete chloroplast genomes in the family Orchidaceae. The bootstrap values were based on 1000 replicates.
